# Interplay between spin proximity effect and charge-dependent exciton dynamics in MoSe_2_/CrBr_3_ van der Waals heterostructures

**DOI:** 10.1038/s41467-020-19816-4

**Published:** 2020-11-26

**Authors:** T. P. Lyons, D. Gillard, A. Molina-Sánchez, A. Misra, F. Withers, P. S. Keatley, A. Kozikov, T. Taniguchi, K. Watanabe, K. S. Novoselov, J. Fernández-Rossier, A. I. Tartakovskii

**Affiliations:** 1 Department of Physics and Astronomy, The University of Sheffield, Sheffield, S3 7RH UK; 2 QuantaLab, International Iberian Nanotechnology Laboratory, Braga, 4715-330 Portugal; 3 School of Physics and Astronomy, The University of Manchester, Manchester, M13 9PL UK; 4 Department of Physics, Indian Institute of Technology Madras (IIT Madras), Chennai, India; 5 Department of Physics and Astronomy, University of Exeter, Exeter, EX4 4QL UK; 6 National Institute for Materials Science, Tsukuba, Ibaraki 305-0044 Japan; 7 Centre for Advanced 2D Materials, National University of Singapore, Singapore, 117546 Singapore; 8 Chongqing 2D Materials Institute, Liangjiang New Area, Chongqing, 400714 China

**Keywords:** Magnetic properties and materials, Semiconductors, Two-dimensional materials

## Abstract

Semiconducting ferromagnet-nonmagnet interfaces in van der Waals heterostructures present a unique opportunity to investigate magnetic proximity interactions dependent upon a multitude of phenomena including valley and layer pseudospins, moiré periodicity, or exceptionally strong Coulomb binding. Here, we report a charge-state dependency of the magnetic proximity effects between MoSe_2_ and CrBr_3_ in photoluminescence, whereby the valley polarization of the MoSe_2_ trion state conforms closely to the local CrBr_3_ magnetization, while the neutral exciton state remains insensitive to the ferromagnet. We attribute this to spin-dependent interlayer charge transfer occurring on timescales between the exciton and trion radiative lifetimes. Going further, we uncover by both the magneto-optical Kerr effect and photoluminescence a domain-like spatial topography of contrasting valley polarization, which we infer to be labyrinthine or otherwise highly intricate, with features smaller than 400 nm corresponding to our optical resolution. Our findings offer a unique insight into the interplay between short-lived valley excitons and spin-dependent interlayer tunneling, while also highlighting MoSe_2_ as a promising candidate to optically interface with exotic spin textures in van der Waals structures.

## Abstract (web summary)

One advantage of van der Waals materials is the ability to combine different materials in layers to form new heterostructures. Here, the authors investigate heterostructures of CrBr_3_ and MoSe_2_, and find that the ferromagnetism of CrBr_3_ enhances the valley dependent optical response of the MoSe_2_.

## Introduction

Few of the materials which have underpinned solid-state physics research over the past century now lack a 2-dimensional crystalline analog^1,2^. This is keenly exemplified by recent discoveries of long-range magnetic order persisting into the monolayer limit in 2-dimensional van der Waals materials^3–7^. However, the true potential of layered crystals lies not simply in having access to a selection of atomically thin proxies for conventional materials, but rather the ability to easily stack and combine these different materials into arbitrarily designed van der Waals heterostructures^8^. Not only are such composites free of dangling bonds and growth-related lattice matching constraints, but also exhibit cumulative and hybridized properties superior to those of the constituent layers, and as such represent the building blocks for future generations of nanoscale information technologies^9–14^.

In this context, the discovery of 2-dimensional magnets in particular realizes a step-change for the nascent field of wholly van der Waals based spintronics^5,15–22^. One such material currently experiencing a resurgence of interest is chromium tribromide (CrBr_3_)^7,23–26^, a layered ferromagnetic semiconductor with Curie temperature 37 K and magnetization 3.85*μ*_*B*_ per Cr atom along the easy *c*-axis^27^. Recent work suggests that, in stark contrast to few-layer CrI_3_ and CrCl_3_ flakes which both exhibit antiferromagnetic interlayer ordering^4,22,28^, the interlayer exchange in exfoliated CrBr_3_ is ferromagnetic, although can depend on stacking order^7,23,24,26^.

The incorporation of this emerging family of magnetic materials with optically active transition metal dichalcogenides (TMDs) is expected to combine the advantageous chiral optical selection rules and spin-valley locking of TMDs with the highly correlated and field-responsive long-range ordering inherent to magnets^17,29–31^. Proximity effects between monolayer TMDs and van der Waals ferromagnets have so far manifested as enhanced valley Zeeman splitting and/or modifications to the photoluminescence (PL) intensity^22,24,30,32^. Where changes in the circular polarization degree of PL are reported, significantly broadened linewidths preclude the extraction of useful spectral information, and individual excitonic states cannot be resolved^30^.

In this work, we study a van der Waals interface formed between the TMD molybdenum diselenide (MoSe_2_) and ferromagnetic CrBr_3_. While the interlayer band alignment is nominally type-II, with the global conduction band minimum found in CrBr_3_, a strong spin polarization of the CrBr_3_ conduction bands gives rise to spin-dependent interlayer charge transfer rates for electrons tunneling from MoSe_2_ to CrBr_3_. This spin-dependent non-radiative decay channel causes a valley-dependent quenching of MoSe_2_ photoluminescence intensity, leading to a strongly enhanced degree of circular polarization (DOCP). Crucially, the DOCP is observed only in the MoSe_2_ trion state, rather than the neutral exciton, thereby providing insight into the interlayer tunneling timescales, which we infer to lie between the exciton and trion radiative lifetimes. By performing wide-field polar Kerr microscopy, we both confirm that the MoSe_2_ trion DOCP is an accurate indicator of local CrBr_3_ magnetization, and gain information about the characteristic magnetic domain length-scales in our sample. Our findings shed new light on the mechanisms of interlayer charge transfer in TMD/magnet van der Waals interfaces. Moreover, our work highlights the importance of optical resolution when studying this new class of layered magnetic materials.

## Results

### MoSe_2_/CrBr_3_ van der Waals heterostructures

**Fig. 1:**
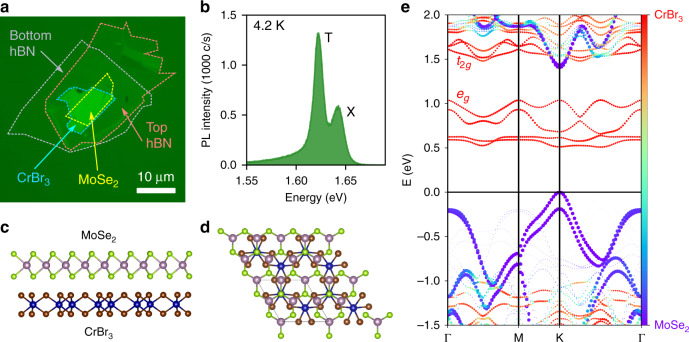
MoSe_2_/CrBr_3_ van der Waals heterostructures. **a** Microscope image of the sample in this work showing multi-layered CrBr_3_, monolayer MoSe_2_, and hBN encapsulation layers. **b** Photoluminescence spectrum from the sample under continuous-wave laser excitation at 1.946 eV and a sample temperature of 4.2 K. Neutral exciton (X) and trion (T) peaks are visible. **c, d** Schematics of a MoSe_2_/CrBr_3_ heterobilayer structure used in the density functional theory calculations, viewed from the side (c) and top (d), where the supercell is highlighted. Here, the experimental lattice constant of monolayer MoSe_2_ is applied to both layers, in order to simplify the calculations, along with setting the relative crystallographic twist angle to zero between the layers (see Supplementary Note 1). Crystal images were produced using VESTA software^33^. **e** The DFT calculated electronic band structure of the MoSe_2_/CrBr_3_ heterobilayer, projected on the host material. The global valence band maximum is localized in MoSe_2_ at *E* = 0. The green band color indicates hybridization between the two materials. The *e*_*g*_-orbitals have spin polarization opposite to the *t*_2*g*_-orbitals.

The sample consists of a monolayer of MoSe_2_ placed directly on top of  ~7–8 nm thick multilayered CrBr_3_, with few-layer hBN encapsulating the structure on both sides, as shown in Fig. 1a. The hBN encapsulation is necessary here owing to the extreme environmental sensitivity of exfoliated CrBr_3_, a property shared also with CrI_3_, in which degradation occurs rapidly under exposure to air and moisture, in a reaction catalyzed by light^34^. The residue-free transfer techniques used during fabrication (see Methods) ensure full contact between the MoSe_2_ and CrBr_3_, such that trapped contaminants do not inhibit the interlayer charge transfer and proximity induced magnetic exchange effects. Photoluminescence (PL) from the sample at 4.2 K under non-resonant optical excitation at 1.946 eV displays a spectrum quite characteristic of MoSe_2_, with a high energy peak ascribed to the neutral exciton (X) and a lower energy peak attributed to the charged exciton, or trion (T) (Fig. 1b)^35,36^. However, the emission intensity from the sample is far lower than typical for exfoliated MoSe_2_, indicating a quenching of PL due to loss of photocarriers from MoSe_2_ into CrBr_3_, reducing the quantum yield.

Density functional theory (DFT) calculations of the electronic band structure of a MoSe_2_/CrBr_3_ heterobilayer (Figs. 1c, d) indicate a type-II interlayer band alignment, with the global conduction band (CB) minimum located in the CrBr_3_ layer, as shown in Fig. 1e. As the material bandgaps depend sensitively on ligand electronegativity, the exact interlayer band alignment at the interface depends heavily on the elemental compositions of each layer^37^ (For details of the DFT calculations see Supplementary Note 1). Photogenerated electrons are predicted to scatter from the MoSe_2_ CB into the CrBr_3_ CB, introducing an additional non-radiative decay channel, corroborating with the observed suppressed luminescence intensity. Such interlayer charge transfer may be aided by electronic interlayer hybridization between the two materials, seen in Fig. 1e as green-shaded regions. Any instances of hybridization would be expected to lead to efficient interlayer charge transfer, via elastic scattering events. Additionally, this heterostructure band alignment suggests that a static electron doping cannot exist in the MoSe_2_ layer, while holes will accumulate in the MoSe_2_ valence band (VB) as electrons tunnel out of the CB non-radiatively. Therefore, we attribute the observed trion luminescence to positively charged excitons, whereby neutral excitons bind to resident holes in the MoSe_2_ VB^30,35^.

A crucial feature of our experiment is that our laser, at 1.946 eV, lies directly in an absorption minimum of CrBr_3_^38–40^. In this way, we ensure that photoexcitation occurs only in MoSe_2_, and so no electron-hole pairs are created in CrBr_3_. Therefore, with the exception of possible electrostatic discharge when the interface is first formed during sample fabrication, we can confidently neglect appreciable hole tunneling from CrBr_3_ to MoSe_2_. As confirmation that our laser is not being absorbed, we search for direct CrBr_3_ luminescence, reported at 1.35 eV under 1.77 eV illumination^25^. Despite rigorous searching, we observe nothing but MoSe_2_ PL between the laser energy and the limit of our detector sensitivity, ~1.1 eV, consistent with CrBr_3_ being transparent to our laser.

The calculated electronic band structure in Fig. 1e also reveals a strong exchange splitting of the CrBr_3_ CB, which is comprised of Cr^3+^*d*-orbitals. Bands arising from the majority-spin *e*_*g*_ orbitals can be seen in red between 0.5 and 1 eV, while the minority-spin *t*_2*g*_ orbitals form bands at 1.5 eV and above^41,42^. Importantly, below the CrBr_3_ Curie temperature  ~ 37 K, these two sets of bands are oppositely spin-polarized out of the sample plane. This is intrinsically tied to the local CrBr_3_ magnetization, such that when the magnetization is flipped between pointing up or down out of the plane, the spin polarization of these two sets of bands reverses. Since the *e*_*g*_ orbitals constitute the majority-spin, they are spin-*↑* (spin-*↓*) when the local CrBr_3_ magnetization points upwards (downwards) out of the sample plane, while the *t*_2*g*_ orbitals are spin-*↓* (spin-*↑*).

Although our sample is not a heterobilayer, but rather multi-layered CrBr_3_, we expect any magnetic proximity effects to arise primarily from the topmost CrBr_3_ layer, owing to the very short range of exchange interactions and interlayer orbital wavefunction overlap^22,43^. We confirm this by calculating and comparing the band structures of MoSe_2_/1L CrBr_3_ and MoSe_2_/2L CrBr_3_, and observe only negligible differences between the two (see Supplementary Note 2).

### Polarization resolved magneto-photoluminescence

**Fig. 2:**
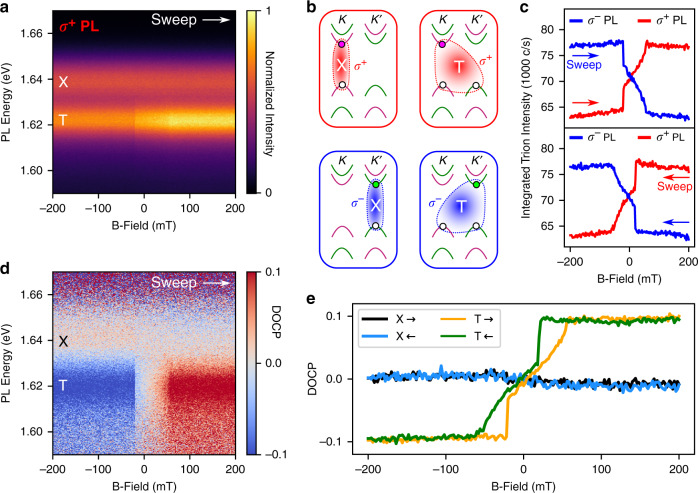
Magneto-photoluminescence of MoSe_2_/CrBr_3_ heterostructures. **a** Photoluminescence (PL) intensity in *σ*^+^ circular polarization as a function of photon energy and external *B*-field, in the forward sweep direction. The neutral exciton (X) and trion (T) are labeled. **b** Schematics of the electron-hole valley configurations of the two ground-state optically bright neutral excitons and positive trions in monolayer MoSe_2_, which emit either a *σ*^+^ or *σ*^−^ polarized photon upon recombination. The pink (green) electron is spin *↑* (*↓*), and the hole is white, regardless of spin. Spin-valley locking ties the electron spin to the emission polarization. **c** Integrated trion PL intensity in both circular polarizations, for the forward (upper panel) and backward (lower panel) *B*-field sweep directions. **d** Degree of circular polarization (DOCP) as a function of photon energy and *B*-field, in the forward sweep direction. **e** DOCP of the exciton and trion states calculated from integrated intensities, shown for both sweep directions, revealing hysteresis type behavior in the trion. The error in DOCP for this figure is  ~ ±0.005.

In order to gain insight into the valley pseudospin dynamics in our sample, we detect PL in *σ*^+^ and *σ*^−^ circular polarizations while sweeping an external magnetic field (*B*) perpendicular to the sample plane, within the range *B* = ±200 mT, in both forward (negative to positive) and backward (positive to negative) sweep directions, at 4.2 K. A *B*-field orientation upwards (downwards) out of the plane is positive (negative). Fig. 2a shows the *σ*^+^ polarized PL as a function of emitted photon energy and *B*-field, in the forward sweep direction. As can be seen, the trion intensity sharply increases at *B* = −20 mT, followed by a steady further increase, until the intensity reaches a plateau at *B* ~ 50 mT. This striking response must be attributed to the influence of CrBr_3_, as the PL intensity of an isolated MoSe_2_ monolayer would change only negligibly under the application of such weak external *B*-fields as used here^44,45^. In stark contrast, the exciton emission, notably distinct from the trion, exhibits no observable change in intensity.

Bright neutral excitons are comprised of an electron-hole pair located in either the K or K’ valley, corresponding to the *σ*^+^ or *σ*^−^ polarization state of the emitted photon (Fig. 2b)^29^. To form a positively charged trion, the exciton binds to an additional hole in the opposite valley (Fig. 2b)^35,46^. This intervalley ground state is highly favored over the intravalley trion configuration, for which the large  ~200 meV spin-orbit coupling strength in the VB is prohibitive^35,46–48^. The electron spin is tied to the emission helicity owing to optical selection rules and spin-valley locking^29,46^.

To elucidate the trion magneto-optical response, we plot the integrated trion intensity in both circular polarizations, in both forwards and backwards *B*-field sweep directions, as shown in Fig. 2c. As can be seen, the trion intensities in opposite polarizations behave exactly symmetrically, such that when the intensity of one polarization decreases the other increases by the same amount. In both sweep directions, the intensities invert as *B* passes through zero, indicating a clear reversal of trion valley populations when very small *B*-fields are applied. A specific response to the sweep direction which is symmetric about zero-field, as seen here, is a hallmark of ferromagnetism, confirming the influence of CrBr_3_ on the MoSe_2_ trion state valley polarization.

A powerful tool to investigate the valley pseudospin dynamics in the sample is the DOCP, $({I}_{{\sigma }^{+}}-{I}_{{\sigma }^{-}})/({I}_{{\sigma }^{+}}+{I}_{{\sigma }^{-}})$, of the emitted luminescence. Fig. 2d presents the DOCP as a function of photon energy and applied *B*-field, in the forward sweep direction. Here, a very prominent trion switching can be seen between robust positive and negative DOCP, while the exciton state remains apparently insensitive to any interactions with CrBr_3_. Plotting the DOCP using integrated intensities, in both sweep directions, as shown in Fig. 2e, brings the sweep direction-dependent response into sharp focus. While the exciton remains at zero, the trion state follows a sweep-dependent path when switching between the two valley polarized extremes, revealing the existence of two hysteretic lobes in the DOCP. We have fabricated and studied an additional 2 samples, both of which reproduce the trion DOCP sensitivity and exciton insensitivity observed in sample 1 (See Supplementary Note 3). Furthermore, by heating the sample under a fixed applied *B*-field, we observe the DOCP to tend to zero above the reported Curie temperature of CrBr_3_,  ~ 37 K (See Supplementary Note 4)^27^.

### Spin-dependent interlayer charge transfer

Ostensibly, an enhanced DOCP in MoSe_2_ trion luminescence indicates a finite valley polarization in the resident carrier population. However, in this case, a hole valley polarization may be ruled out as the origin of the observed trion DOCP for three reasons. Firstly, any imbalance between the itinerant carrier populations of the two valleys leads not only to a change in trion intensity, but also to an opposite and proportional change in exciton intensity, owing to the intervalley nature of the trion ground state^44^. We observe no corresponding change in exciton intensity in Fig. 2a, and no mirroring of the trion DOCP by the exciton DOCP in Figs. 2d and e. Secondly, the observed trion DOCP cannot be due to a suppression of intervalley relaxation, as the sign of the DOCP is independent of the polarization state of the laser, and so the system retains no memory of optical valley initialization (see Supplementary Note 5). Thirdly, our DFT calculations predict a negligible proximity induced spin splitting in the MoSe_2_ VB maxima, thereby precluding the generation of a static valley polarization in the itinerant hole population in thermal equilibrium. The band dependence of the proximity induced spin splitting is ultimately related to their different orbital content, with the VB orbitals being oriented preferably in the molybdenum plane, and so experiencing a weak proximity effect. The orbitals constituting the MoSe_2_ CB edges do protrude more out of the molybdenum plane, and so do experience a modest spin splitting due to proximity, which we measure to be <0.5 meV (Supplementary Notes 1 and 6). Regardless, this splitting is in the CB and so cannot generate a static hole valley polarization in the VB.

**Fig. 3:**
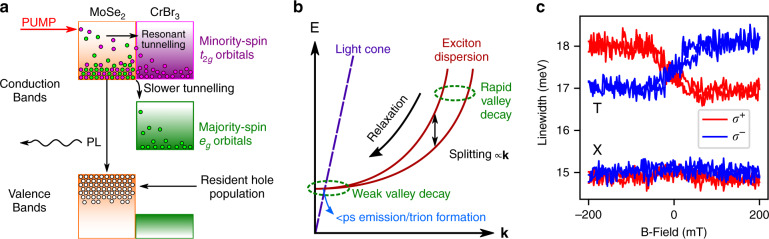
Spin-dependent interlayer tunneling. **a** Schematic of spin-dependent interlayer charge transfer over the MoSe_2_/CrBr_3_ interface. The type-II band alignment precludes a static electron doping in MoSe_2_, and so we infer a resident hole population. In CrBr_3_, the CB is strongly split into oppositely spin-polarized bands. Only the minority-spin bands are resonant with the MoSe_2_ CB. Upon optical injection of electron-hole pairs, the electrons may tunnel from MoSe_2_ to CrBr_3_, with efficiency depending on their spin (green or purple indicate opposite spin). As CrBr_3_ is transparent at our laser energy, absorption is negligible, and so we expect no appreciable dynamic hole transfer from CrBr_3_ to MoSe_2_. **b** Diagram of exciton dispersion in MoSe_2_, with an energy splitting (the L-T splitting, see main text) proportional to in-plane wavevector **k**. The L-T splitting acts as an effective magnetic field inducing valley pseudospin relaxation in the presence of disorder scattering. Outside the light cone, efficient valley depolarization prevents spin-dependent tunneling from influencing the eventual DOCP. At low **k**, the spin-dependent tunneling is able to influence the DOCP observed in PL. At vanishing center of mass momentum, the exciton population either radiatively recombines (sub-ps) or else binds to a resident hole to form a long-lived trion valley state, which will be susceptible to spin-dependent tunneling. **c** Polarization resolved PL linewidths of the exciton (X) and trion (T). The trion linewidth shows opposing behavior in different polarizations, indicating that the lifetime is influenced by spin-dependent interlayer tunneling. The exciton has a constant linewidth, indicating insensitivity to CrBr_3_ magnetization.

Therefore, rather than a static hole valley polarization, we attribute the observed DOCP to spin-dependent interlayer electron transfer, which translates to valley-dependent charge leakage from MoSe_2_ owing to the spin-valley locking effect. Similar results have previously been observed in conventional ferromagnet-quantum well systems^49^. The particular interlayer band alignment in this structure results in the MoSe_2_ CB being resonant only with the minority-spin (*t*_2*g*_) bands of CrBr_3_, while the majority-spin (*e*_*g*_) bands are non-resonant by several hundred meV (Fig. 1e). Therefore, we expect that photogenerated electrons in the MoSe_2_ CB will be able to tunnel into the CrBr_3_ CB more efficiently if they are spin aligned with the CrBr_3_ minority-spin states. In order to tunnel into the majority-spin bands, multiple phonon scattering or high energy Auger-like interactions would be required, resulting in poor tunneling efficiency (Fig. 3a). We also note the aforementioned lack of absorption of our laser by CrBr_3_, which ensures that dynamic hole transfer (spin-dependent or otherwise) from CrBr_3_ into MoSe_2_ can safely be neglected. Consequently, we can be confident that the observed trion DOCP arises purely from electron tunneling from MoSe_2_ into CrBr_3_, which introduces an additional non-radiative decay for K or K′ valley trion states, depending on the CrBr_3_ magnetization.

We note that our PL signal is comparably quenched in both *σ*^+^ and *σ*^−^ polarizations, yet shows a 10% DOCP in the trion state. We attribute this to a complex interplay between exciton valley dynamics and spin-dependent interlayer charge transfer in our sample. In MoSe_2_, the exciton dispersion experiences an energy splitting between excitons with dipole moment parallel (longitudinal, L) or perpendicular (transverse, T) to their in-plane wavevector, **k**, arising from the long range electron-hole exchange interaction^50,51^. This L-T splitting is linearly proportional to **k** outside the light cone, and manifests as an effective magnetic field acting on the exciton valley pseudospin, causing precession and associated valley depolarization, aided by disorder scattering^50–52^. Consequently, valley depolarization is extremely efficient (<0.1 ps^53^) for high-**k** excitons, and suppressed for low-**k** excitons (Fig. 3b). As such, any valley polarization from interlayer tunneling at higher energies (closer to the laser at 1.946 eV) is quickly erased, and the tunneling serves only to uniformly quench both the *σ*^+^ and *σ*^−^ PL spectra. We expect the tunneling to be very efficient at these higher energies owing to resonant interlayer band alignment and regions of hybridization evident in Fig. 1e between the MoSe_2_ CB and CrBr_3 _*t*_2*g*_ states. This efficient hot electron tunneling will indeed be highly spin polarized, but the comparably fast valley depolarization in MoSe_2_ ensures that no signatures persist in the observed DOCP.

We therefore believe that the measured DOCP is sensitive only to spin/valley dynamics in the energy range corresponding to vanishing exciton center-of-mass momentum, close to or inside the light cone. At these low wavevectors, the exchange induced valley depolarization is weak, allowing spin-dependent tunneling to influence the DOCP in luminescence. The fact that DOCP is observed only in the trion state implies that the additional non-radiative decay arising from charge transfer in this low energy range must be slower than the exciton radiative lifetime (sub-ps^54^), but faster than the lifetime of the trion state, which is an order of magnitude longer (>10 ps)^55^. Our conclusion is supported by analysis of the PL linewidths, revealing a magnetization dependent change in the trion linewidth of  ~1 meV, while the exciton linewidth remains constant (Fig. 3c). This allows us to estimate that the additional non-radiative decay experienced by the trion owing to spin-dependent charge transfer at these lower energies is ~0.7 ps. The tunneling efficiency is generally influenced by various factors, including band hybridization, and energy or momentum offsets^9,56–59^. Our calculations (Fig. 1e) predict that at the bottom of the MoSe_2_ CB there is a low degree of hybridization with CrBr_3_ CB states, and the energy and momentum offsets for possible electron scattering are large. These factors can explain the relatively long charge transfer times deduced from the experiment. We note that the tunneling rates may vary over a large range, as was revealed in recent pump-probe experiments on twisted TMD heterobilayers, where sub-ps to 5 ps tunneling times were observed, drastically dependent on the relative crystallographic twist angle between the layers^57^.

In stark contrast to MoSe_2_, the valley pseudospin lifetime in WSe_2_ exceeds the PL lifetime^60^, and so the final PL DOCP will be influenced by the full extent of spin-dependent interlayer tunneling occurring between the laser energy and the PL energy. This explains why the reported DOCP in WSe_2_/CrI_3_ structures is larger than our MoSe_2_ case, while also explaining why we observe no dependence on laser polarization in our sample (Supplementary Note 5), whereas results from WSe_2_/CrI_3_ structures depend fundamentally on the laser polarization^30^.

### Domain-like valley polarization topography

The intertwined relationship between trion PL and the local magnetization of CrBr_3_, as demonstrated in Fig. 2e, means that the DOCP serves as a proxy magnetization or M-H curve. Viewed in this way, the trion polarization degree bears all the hallmarks of the magnetization response of a thin ferromagnetic film with perpendicular magnetocrystalline anisotropy, in which the magnetization of each domain must be aligned upwards or downwards out of the plane^7,25,61^. When ∣*B*∣ > *B*_*sat*_ = 50 mT, plateaus are observed, corresponding to magnetic saturation parallel to the external field, whereby the CrBr_3_ is essentially in a single magnetic domain state. At field strengths lower than the saturation field, a complex response is observed featuring two marked hysteretic lobes, with a smooth linear gradient around *B* = 0. Such a dependence is purely the result of magnetic domain dynamics, whereby the two discontinuities at *B* = ±20 mT appear as a result of spontaneous magnetic nucleation^25,61^. This process occurs when a saturating *B*-field is gradually removed, until a balance is tipped in favor of the formation of antiparallel adjacent domains, representing a lower energy configuration. For the metastable saturation state to break down, a potential barrier associated with domain wall formation must be overcome^61^.

At very low fields, ∣*B*∣ < 20 mT, the average magnetization responds linearly to *B* owing to unopposed lateral domain wall motion through the flake, allowing domains to grow and shrink as appropriate. As the CrBr_3_ flake is a single crystal, domain wall motion ought to be generally unobstructed, in contrast to polycrystalline thin-film ferromagnets, in which grain boundaries may impede domain growth^62^. Introducing certain defects and disorder to the flake may also lead to domain wall pinning which opens up a hysteresis loop at zero field, consistent with the more typical remanence signatures of conventional ferromagnets^4,30^. As such, the absence of coercivity in this sample indicates both a weak anisotropy^24^, and a low density of defects of the type which may impede lateral domain wall motion^61,63^. However, we note that there may be a high density of other defects which have a weaker influence on domain dynamics, consistent with the unstable nature of CrBr_3_ (see Supplementary Note 8).

**Fig. 4:**
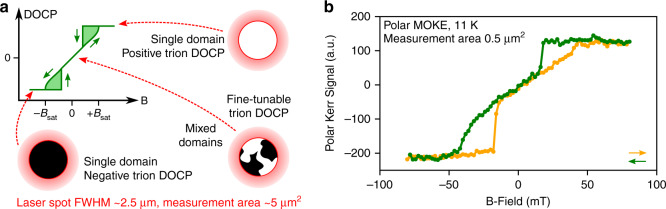
Domain-like arrangement of trion valley polarization. **a** Illustration of the trion degree of circular polarization (DOCP), with indications of the spatial arrangement of trion valley polarization at various points on the curve. Above ∣*B*_*sat*_∣, the CrBr_3_ is in a single domain state and there is a uniform spin preference for electron tunneling from MoSe_2_ to CrBr_3_. At lower *B*-fields, the relative strengths of spin *↑* or *↓* tunneling may be fine-tuned by control of the external field, which determines the relative spatial area occupied by adjacent domains. The red circles indicate an approximation of the laser spot area in the PL study, about 5 μm^2^, over which the domain behavior is averaged. **b** Polar magneto-optical Kerr effect (MOKE) signal from the same sample, with a much smaller measurement area of 0.5 μm^2^. The domain nucleation behavior is retained, indicating a very small length scale of domains.

The crucial consequence of these CrBr_3_ domain dynamics is that the spin-dependent interlayer charge transfer in the heterostructure adopts a spatial topography and magnetic response describable by the physics of ferromagnetic domains. As shown in Fig. 4a, when *B* > *B*_*sat*_ (*B* < −*B*_*sat*_), the charge transfer is preferentially spin-*↓* (spin-*↑*) over the entire interface. However, when ∣*B*∣ < *B*_*sat*_, the interlayer charge transfer rates adopt a highly contrasting spatial arrangement over the sample, which in turn creates domain-like regions of opposite valley polarization co-existing in the MoSe_2_ trion population. Cumulatively, a reversal of the external *B*-field gives efficient selectivity between opposite preferential tunneling rates for electrons of spin-*↑* or spin-*↓*, while control of the field strength close to zero allows fine-tuning of their relative prevalence.

We note that in our PL experiments, we measure an average of domain activity within the area of our laser spot, ~5 μm^2^. In order to gain insight into the characteristic length scales of the trion valley polarization domains in our sample, we measure the local magnetization via the polar magneto-optical Kerr effect (MOKE) in an optical microscope utilizing broadband illumination and wide-field high spatial resolution detection (Fig. 4b). We expect that the polar Kerr signal arises mainly from the CrBr_3_ layers of the sample, given the maximum of the illumination energy at 2.7 eV closely matches the CrBr_3_ main absorption resonance at 2.5 eV^37^. Therefore, the close agreement between the PL from MoSe_2_ and the Kerr signal indicates a robust proximity effect and strong layer interaction^64^. Despite our MOKE measurement area being  ~0.5 μm^2^, an order of magnitude smaller than in the PL experiment, there is close agreement between the two magneto-optical techniques, indicating that the domains are significantly smaller than the length scales of our optical probes. Indeed, no domain activity could be resolved in the Kerr microscope at all, confirming a domain size smaller than the optical resolution of the system,  ~400 nm (See Supplementary Note 8). High spatial resolution techniques such as scanning probe magnetometry would be required to resolve the domain structure in our sample^5,65^.

Our findings are in good agreement with previous work on CrBr_3_ flakes in which electron microscopy reveals stripe domains with widths between 20 and 200 nm^66^. In thin-film ferromagnets such as our CrBr_3_ flake, the domain size is governed by a complex interplay between magnetocrystalline and shape anisotropies, domain wall width, in-plane remanence, exchange energy, and magnetostatic interaction energy between the top and bottom film surfaces^67^. The domain dynamics will therefore depend sensitively on the specific balance of these parameters, leading to significant variation. Indeed, complex and diverse domain patterns have been observed in CrBr_3_ including stripe, honeycomb, bubble, and cone domain structures^68^. Weak in-plane external *B*-field orientation, disorder, temperature, or unpredictable local fluctuations, can all influence the domain patterns formed during each *B*-field cycle, with no correlation expected between patterns present before and after each instance of saturation, unless external input is used to provide control^68^. We note that the trion Bohr radius in MoSe_2_ ( ~1 nm), is 1−2 orders of magnitude smaller than the domain sizes inferred by our observations. This suggests that control of domain formation dynamics in CrBr_3_, by the techniques mentioned above, would generate desired spatial patterns of contrasting electronic and trionic spin-valley polarization in MoSe_2_, be they stripe, honeycomb, bubble, and so on, with important consequences for opto-spintronic device design at the nanoscale.

## Discussion

To conclude, we observe spectrally dependent magnetic proximity effects in photoluminescence from monolayer MoSe_2_ in contact with few-layer CrBr_3_. The local out of plane magnetization in CrBr_3_ is imprinted in the MoSe_2_ trion state valley polarization, while the neutral exciton state bears no hallmarks of proximity interactions. We attribute the observed magneto-optical response to a spin-dependent interlayer charge transfer process, whereby electrons tunnel from the CB of MoSe_2_ into the CB of CrBr_3_ with differing rates depending on their real spin. This leaves the optically pumped exciton population in MoSe_2_ subject to competing mechanisms of spin-dependent tunneling and valley pseudospin depolarization, ultimately leading to the observed charge-state dependency of the proximity effects in photoluminescence. Furthermore, we infer that in the absence of an applied *B*-field, the sample displays simultaneous manifestation of positive and negative valley polarization in the trion population, with an intricate spatial topography conforming to the magnetic domains of CrBr_3_.

Our results demonstrate a highly localized (<400 nm) technique to engineer the interlayer tunneling rates of electrons in van der Waals heterostructures. The wide-ranging implications of this extend to, for example, incorporation of a layered ferromagnet beneath a TMD heterobilayer to enable control of the interlayer exciton lifetime, or local spin and charge densities, serving as a powerful complementary technique to the design of the crystallographic twist angle. Such enticing avenues of band structure engineering are yet to be explored. A further tantalizing possibility is the mimicry of unconventional spin textures by 2-dimensional semiconductors in proximity to magnetic materials. The local sensitivity of excitonic valley polarization may be extended to employ monolayer MoSe_2_ as an atomically thin optical probe for such exotic phenomena as skyrmions or domain wall racetrack devices, thus realizing a wealth of far-reaching consequences along the boundary between exciton physics and van der Waals spintronics.

## Methods

### Low-temperature magneto-optical spectroscopy

Low-temperature magneto-photoluminescence spectroscopy was performed by mounting the sample in a liquid helium bath cryostat at 4.2 K with free-space optical access and a superconducting magnet coil. Non-resonant continuous-wave excitation at 1.946 eV in either *σ*^+^ or *σ*^−^ circular polarization was used, along with helicity selective circularly polarized PL collection, directed through a 0.75 m spectrometer and onto a nitrogen-cooled high sensitivity charge-coupled device. The helicity of the laser has no influence on the results measured, as discussed in Supplementary Note 5. To obtain linewidths and valley Zeeman splitting of exciton and trion peaks, Gaussian peak fitting was carried out on the PL spectra, as discussed in Supplementary Note 6.

### Density functional theory calculations

See Supplementary Notes 1 and 2.

### Low-temperature wide field Kerr microscopy

Spatially resolved polar Kerr hysteresis loops were acquired at low temperature using a wide field Kerr microscope equipped with a small helium gas flow cryostat designed for microscopy. A long working distance, high numerical aperture (NA 0.7) objective lens was used to achieve a spatial resolution of 400 nm. Spatial stability was achieved by mounting the cryostat on a piezoelectric stage for active drift correction. Hysteresis loops were acquired from user defined microscale regions of interest on the sample. The polar Kerr effect allowed sensitivity to the out-of-plane component of the magnetization in response to a magnetic field as it was swept perpendicular to the sample plane. The sample illumination was linearly polarized, while polarization changes of the reflected light due to the polar Kerr effect were detected as intensity changes using a high sensitivity CMOS camera after the light was passed through a nearly crossed analyzer. A background polarization rotation that was linear in applied field was measured from the sample substrate and subtracted from the acquired loops. The background was due to the Faraday rotation in the objective lens and cryostat window when subject to a field applied parallel to the polar axis. Differential imaging allowed non-magnetic contrast such as sample topography to be subtracted leaving only the contrast due to the out-of-plane component of the sample magnetization.

### Sample fabrication

A heterostructure comprising of MoSe_2_/CrBr_3_ encapsulated within thin hexagonal boron nitride (hBN) layers was assembled on a DBR substrate (top layer  ~100 nm SiO_2_) following standard dry transfer procedures using a poly (methyl methacrylate) (PMMA) membrane. MoSe_2_ crystals were exfoliated on 290 nm SiO_2_/Si substrate and monolayers of MoSe_2_ were identified using optical contrast and room temperature PL measurements. To begin the heterostructure assembly, first, a monolayer of MoSe_2_ was picked up by a thin hBN (~8−10 nm) using the PMMA membrane. CrBr_3_ crystals, purchased from commercial supplier HQ Graphene, were exfoliated on to a 290 nm SiO_2_/Si substrate in an argon-filled glove box maintaining the water and oxygen concentration less than 0.1 ppm. CrBr_3_ flakes of different thicknesses (2–6 layers) could be easily identified using the contrast variation under different color filters and dark-field imaging. However, for this work, a relatively thick  ~7–8 nm thick flake was used. A suitable (uniform and crack free) CrBr_3_ layer was picked up by MoSe_2_/hBN/PMMA membrane in the glove box prepared in the first step. To complete the encapsulation of MoSe_2_/CrBr_3_ between hBN layers, a thin hBN layer was further picked up by this membrane. Finally, the complete stack (hBN/CrBr_3_/MoSe_2_/hBN) on PMMA was dropped onto a DBR mirror by cutting the PMMA membrane. The PMMA was washed away in acetone and in IPA.

## Supplementary information

Supplementary Information

## Data Availability

Experimental data from this work is stored on University of Sheffield central file servers and is available on request by contacting the corresponding authors.
