# Supplementary material for: Interplay between spin proximity effect and charge-dependent exciton dynamics in MoSe2/CrBr3 van der Waals heterostructures
Source: Nat Commun. 2020 Nov 26;11:6021. doi: 10.1038/s41467-020-19816-4 (PMC7691354; doi:10.1038/s41467-020-19816-4)
Supplement: Supplementary file 1 — Supplementary Information [file 41467_2020_19816_MOESM1_ESM.pdf]

Supplementary Information for: Interplay between spin proximity effect and charge-dependent exciton dynamics in $MoSe_2$ / $CrBr_3$ van der Waals heterostructures

T. P. Lyons,[1,*] D. Gillard,[1] A. Molina-Sánchez,[2] A. Misra,[3,4] F. Withers,[5] P. S. Keatley,[5] A. Kozikov,[3] T. Taniguchi,[6] K. Watanabe,[6] K. S. Novoselov,[3,7,8] J. Fernández-Rossier,[2] and A. I. Tartakovskii[1,†]

[1]*Department of Physics and Astronomy, The University of Sheffield, Sheffield S3 7RH, UK*
[2]*QuantaLab, International Iberian Nanotechnology Laboratory, 4715-330 Braga, Portugal*
[3]*School of Physics and Astronomy, The University of Manchester, Manchester M13 9PL, UK*
[4]*Department of Physics, Indian Institute of Technology Madras (IIT Madras), Chennai, India*
[5]*College of Engineering, Mathematics and Physical Sciences, University of Exeter, Exeter, EX4 4QF, UK*
[6]*National Institute for Materials Science, Tsukuba, Ibaraki 305-0044, Japan*
[7]*Centre for Advanced 2D Materials, National University of Singapore, 117546 Singapore*
[8]*Chongqing 2D Materials Institute, Liangjiang New Area, Chongqing, 400714, China*

Supplementary Note 1: Details of the DFT calculations

The *ab initio* calculations of the electronic structure of the heterostructure $MoSe_2/CrBr_3$ have been performed using density-functional theory (DFT) at the level of the local density approximation (LDA), as implemented in Quantum Espresso package [1]. In addition we have applied the on-site Hubbard correction with values $U = 1.5$ eV and Hund's exchange interaction $J = 0.5$ eV [2]. We have included spin-orbit interaction with spinorial wave functions, using norm-conserving full relativistic pseudopotentials. The pseudopotentials of Cr and Mo include semi-core valence electrons and have been generated with ONCVPSP and PSEUDOJO [3, 4]. The electronic density converges with an energy cutoff of 80 Ry and a **k**-grid of $12 \times 12 \times 1$. We use a slab model with a 17 Å vacuum thickness to avoid interactions between periodic images.

In order to make the interpretation of the band structure easier we have followed the unfolding procedure formulated by Popescu and Zunger [5]. This method maps the energy eigenvalues obtained in supercell calculations into an effective band structure (EBS). The reference band structure is that of monolayer $MoSe_2$.

We have performed two sets of *ab initio* calculations: (i) imposing the experimental lattice constant of $MoSe_2$ and relaxing the atomic positions of the whole heterostructure, and (ii) relaxing the lattice parameter of the supercell. In this way we assure that no artifact from the simulation alters the conclusions. Figure 1a-b shows the EBS material-projected and spin-projected, respectively, for the case of $MoSe_2$ experimental lattice constant. We find the strongest hybridization of the EBS at the intermediate Q point in the conduction band. Moreover, the conduction band is immersed within the t_{2g} bands of $CrBr_3$. Figure 1c shows the detail of the conduction band at the −K and +K points of the EBS, which reveals a valley splitting of 2.2 meV. In free-standing $MoSe_2$ and without magnetic field, conduction band states at −K and +K have the same energy. Our calculations indicate no valley splitting in the valence band.

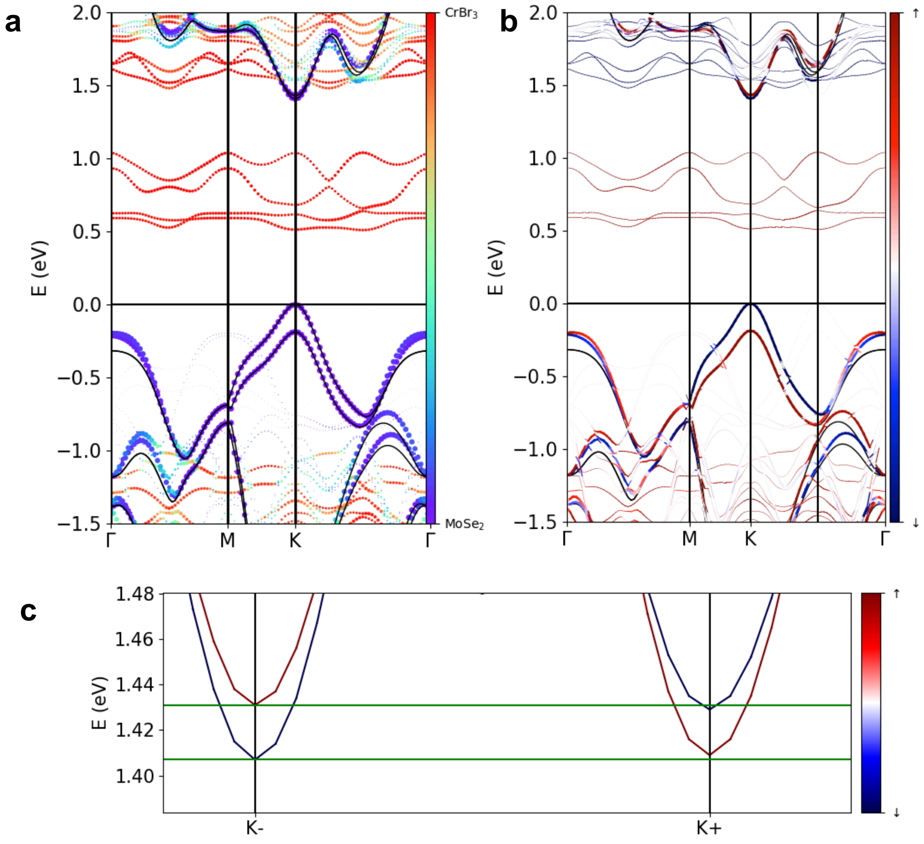

FIG. 1: Effective band structure of $MoSe_2/CrBr_3$ with $MoSe_2$ experimental lattice constant. (a) Material-projected EBS, (b) spin-projected EBS and (c) $MoSe_2$ conduction bands around the ±K points, revealing a valley splitting of 2.2 meV.

Figure 2 shows the relevant wavefunctions at important points in the EBS at the K-point. There is evidence of interlayer band hybridization between the materials' conduction bands, corresponding to box 5 in Figure 2. The orbital content of the $MoSe_2$ valence band is localized primarily in the molybdenum plane (box 1), and so experiences a far weaker spin proximity effect as compared to the $MoSe_2$ conduction band, in which the orbital spread is more prominent out of the plane (boxes 3 and 4). This explains the breaking of valley degeneracy in the conduction band (Figure 1c), absent for the valence band.

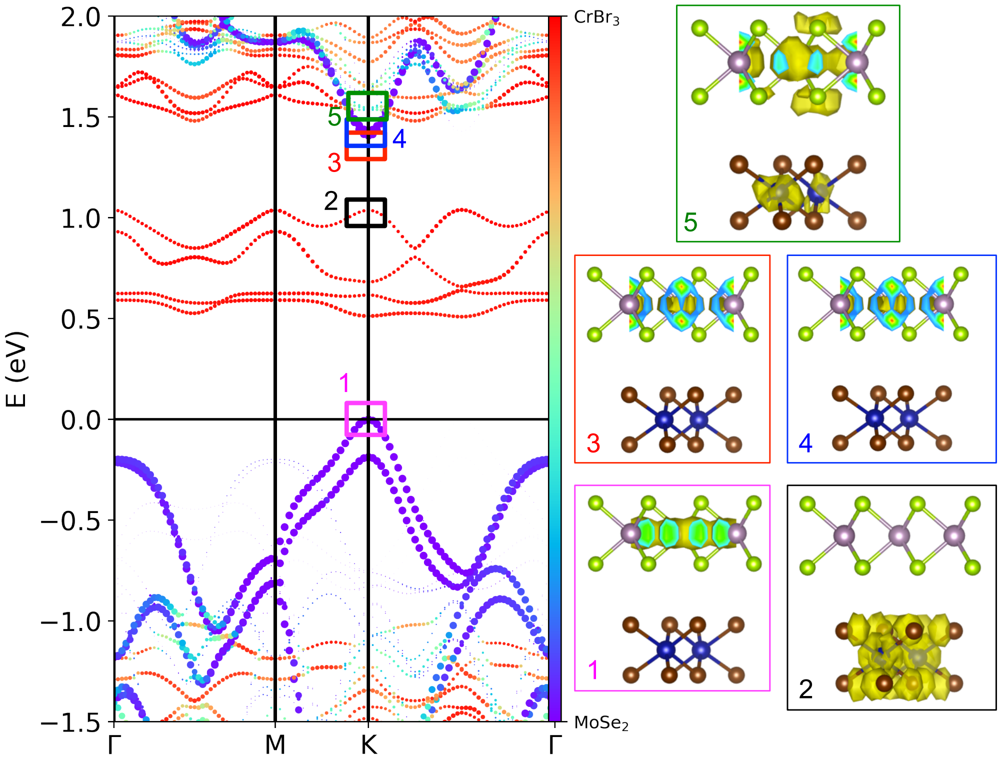

FIG. 2: Band structure of $MoSe_2$ / $CrBr_3$ (as in Fig. 1e of Main Text) together with the wave function density of the following band states: 1. Top of valence band ($MoSe_2$); 2. Conduction band e_g of $CrBr_3$; 3 and 4. Conduction band states of $MoSe_2$ spin up and down; 5. Conduction band state $MoSe_2$-$CrBr_3$.

We have also evaluated the EBS for an optimized heterostructure, as shown in Figure 3. In this case we find an indirect bandgap for $MoSe_2$, and a stronger hybridization of the conduction band at the Q-point. This is a well-known consequence of the dependency of the bandgap directness on the lattice parameter in transition metal dichalcogenides [6]. In this case a conduction band valley splitting is also expected, as shown in Figure 3c.

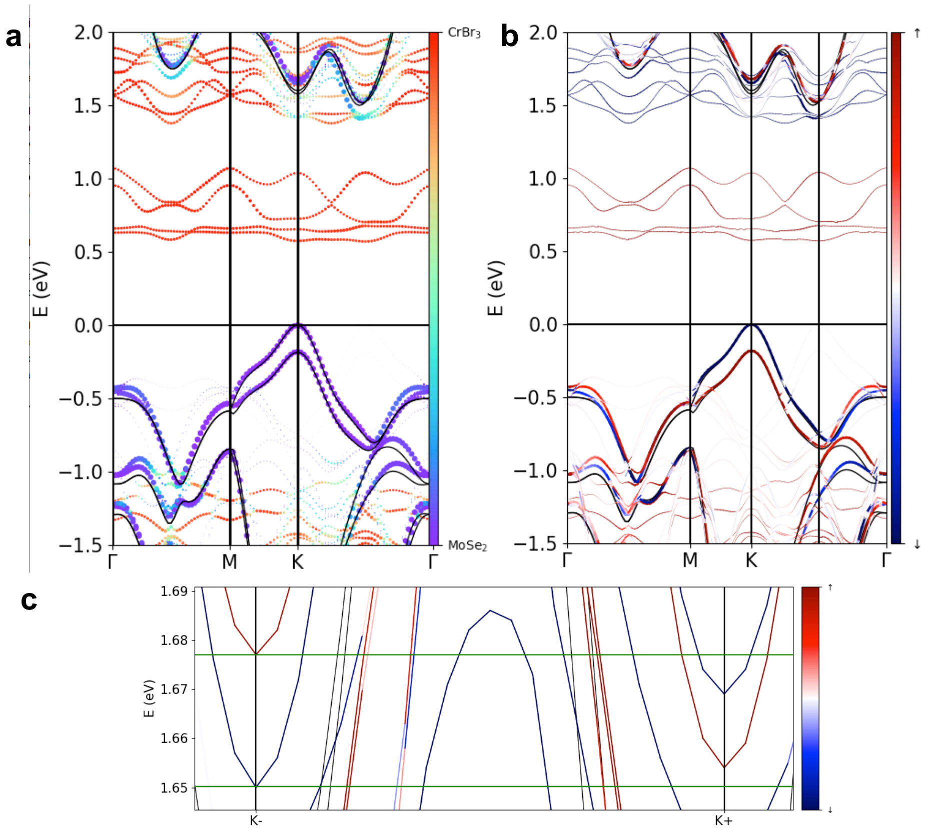

FIG. 3: Effective band structure of $MoSe_2/CrBr_3$ with optimized lattice parameters. (a) Material-projected EBS, (b) spin-projected EBS and (c) conduction bands around the ±K points.

Supplementary Note 2: Band structure of monolayer-$MoSe_2$ on bilayer-$CrBr_3$

We have confirmed the lack of significant contribution to the proximity effects from underlying $CrBr_3$ layers by comparing the electronic structure of monolayer $MoSe_2$ on top of single-layer and double-layer $CrBr_3$. Essentially we find that adding more layers results in the increasing of states from $CrBr_3$.

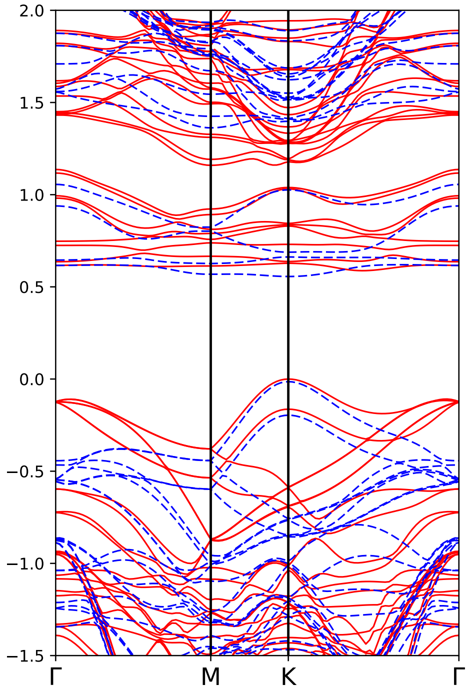

FIG. 4: Comparison of effective band structures for the cases of $MoSe_2$ on top of monolayer $CrBr_3$ (blue dashed lines) or $MoSe_2$ on top of bilayer $CrBr_3$ (red lines). The thicker $CrBr_3$ adds more bands to the EBS as a whole, and the absolute energies of some of the bands shift, but the e_g bands remain at several hundred millielectron Volts below the t_{2g} bands, and so the spin-dependent interlayer tunnelling discussed in the Main Text is not expected to change substantially.

Supplementary Note 3: Measurements on additional samples

We have fabricated and measured the DOCP in photoluminescence of 2 additional $MoSe_2$ / $CrBr_3$ samples. The results are presented below. As can be seen, both samples reproduce the core finding from the sample presented in the Main Text, that is, exciton insensitivity to the proximity effects, alongside trion DOCP switching (evidenced by the sharp discontinuity, present only in the trion state, in the colourmaps below) arising from spin-dependent interlayer charge transfer.

We note some minor differences attributable to sample variation. This is to be expected, especially considering that these additional samples were not fabricated using the same bulk $CrBr_3$ crystal as Sample 1 presented in the Main Text. For instance, the exact shape of the trion DOCP vs B is not identical to Sample 1. This reflects a difference in ferromagnetic domain dynamics. As discussed in the Main Text, the domain formation patterns and sizes depend heavily on a host of factors which will inevitably vary from one flake to the next. We also note that $CrBr_3$ is very unstable and sensitive to degradation. We cannot be sure how many layers within each flake are still magnetically active, and how that may influence the domain dynamics of the flake as a whole.

The other noticeable difference is that the trion DOCP in Sample 3 does not cross DOCP $= 0$. This is a linear offset in DOCP which does not influence the hysteresis behaviour. We expect that it arises from a portion of the $CrBr_3$ flake which is not responsive to B-field, as the magnetization may be pinned by disorder or degradation. In all 3 samples, only the trion state is sensitive to $CrBr_3$ magnetization fluctuations, while the exciton displays only a shallow gradient owing to the conventional valley Zeeman effect, rather than any proximity interactions.

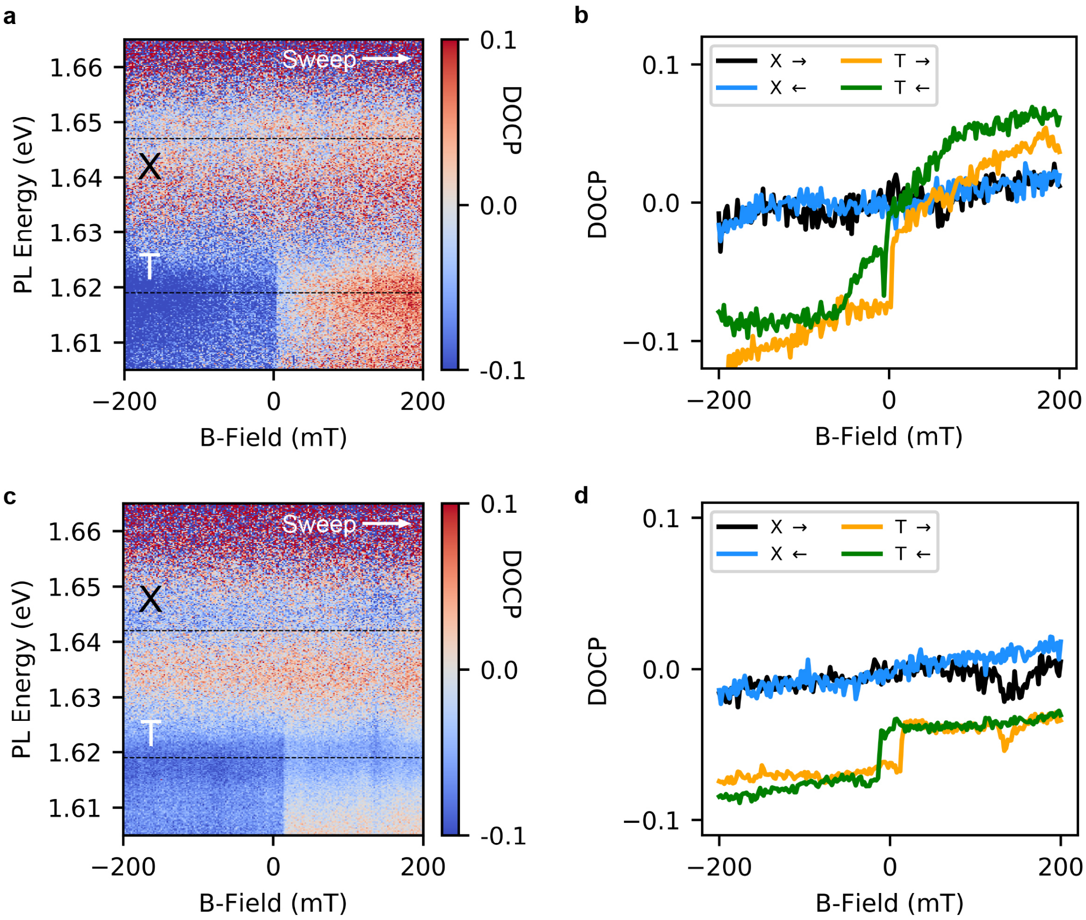

FIG. 5: Data from additional samples. (a,c) Degree of circular polarization (DOCP) from Sample 2 (a) and Sample 3 (c) in the forward sweep direction. (b,d) DOCP of exciton and trion in Sample 2 (b) and Sample 3 (d) calculated using integrated intensities, in both B-Field sweep directions.

Supplementary Note 4: Temperature dependence of trion DOCP

We heat the sample from the base temperature of 4.2 K up to 60 K, under a constant applied magnetic field of $B = +200$ mT, in order to saturate the $CrBr_3$ magnetization. The polarization degree is observed to decrease with increasing temperature, and to tend towards zero above the reported Curie temperature of $CrBr_3$ of $\sim$ 37 K [7]. We note that the $CrBr_3$ becomes paramagnetic above the Curie temperature, and so some spin polarization of the electronic bands is likely to persist above $\sim$ 37 K owing to the influence of the external saturation field. Therefore, no sharp drop in DOCP is necessarily expected exactly at the Curie point.

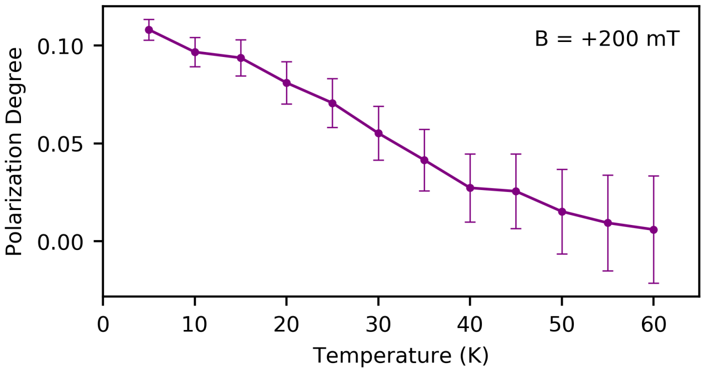

FIG. 6: Trion polarization degree as a function of sample temperature, while heated under a fixed external B-field of strength 200 mT, corresponding to saturation of the $CrBr_3$ magnetization parallel to B. The error in the measurement increases with temperature as the PL signal intensity decreases.

Supplementary Note 5: Insensitivity of results to polarization state of laser

The laser remains in σ^+ circular polarization for all data presented in the Main Text. Nominally, this polarization state addresses only the +K valley of $MoSe_2$, rather than the −K valley, which in principle may introduce a finite valley polarization of photogenerated carriers, excitons or trions, leading to a non-zero circular polarization degree in the eventual photoluminescence. However, the lack of significant retention of non-resonantly optically injected valley polarization in photoluminescence from monolayer $MoSe_2$ is well known [8, 9], most commonly attributed to extremely efficient and rapid depolarization owing to long-range electron-hole exchange interactions, which effectively couple excitons of opposite valley index [10, 11].

To confirm that the polarization state of the laser in our experiments has no effect on the results, we repeat our experiments with σ^- laser polarization, and measure polarization resolved PL intensity, as presented in the Main Text. The result for both laser polarizations are shown in Suppl. Fig. 7, where it is clear that the PL response is essentially identical regardless of laser polarization, confirming that any non-zero polarization in emission is a result of interaction with the ferromagnet or external B-field, and not the laser itself.

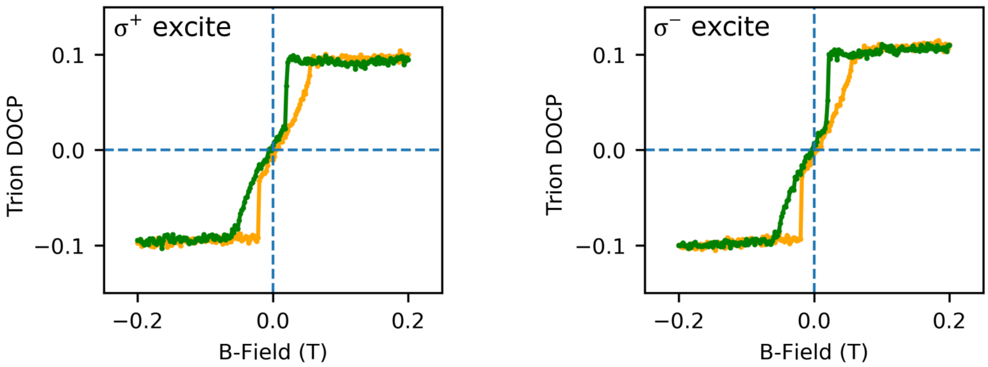

FIG. 7: Comparison of the degree of circular polarization (DOCP, defined in the Main Text) for forward and backward B-field sweep directions, while under either σ^+ (left panel) or σ^- (right panel) excitation laser polarization. The left panel is the same data as shown in the Main Text Fig. 2e. The identical result displayed here confirms that the polarization degree dependence is due to interactions with the $CrBr_3$ and external B-field, rather than the laser polarization, which has no effect owing to extremely rapid valley depolarization. This ensures that all optically generated valley polarization is lost before luminescence, if the excitation is sufficiently non-resonant, as is the case here (laser energy is $\sim$ 300 meV above the exciton energy).

Supplementary Note 6: Peak fitting for extraction of linewidth and valley splitting

In order to extract the linewidths of the exciton and trion states as a function of applied external B-field (as shown in Main Text Fig. 3c), the polarization resolved photoluminescence spectra at each B-field increment were fitted to two Gaussian peak functions, corresponding to X and T. From the fitting we also extract a small valley splitting in both states, attributed to conduction band splitting as discussed in Supplementary Note 1. While the DFT calculations predict a larger proximity induced CB valley splitting of ~ 2 meV, the smaller experimental value may be due to the fact that interlayer exchange couplings are notoriously difficult to compute accurately, the fact that lattice alignment may be different from the one assumed in the calculation, and the fact that excitonic effects have not been included in the calculation.

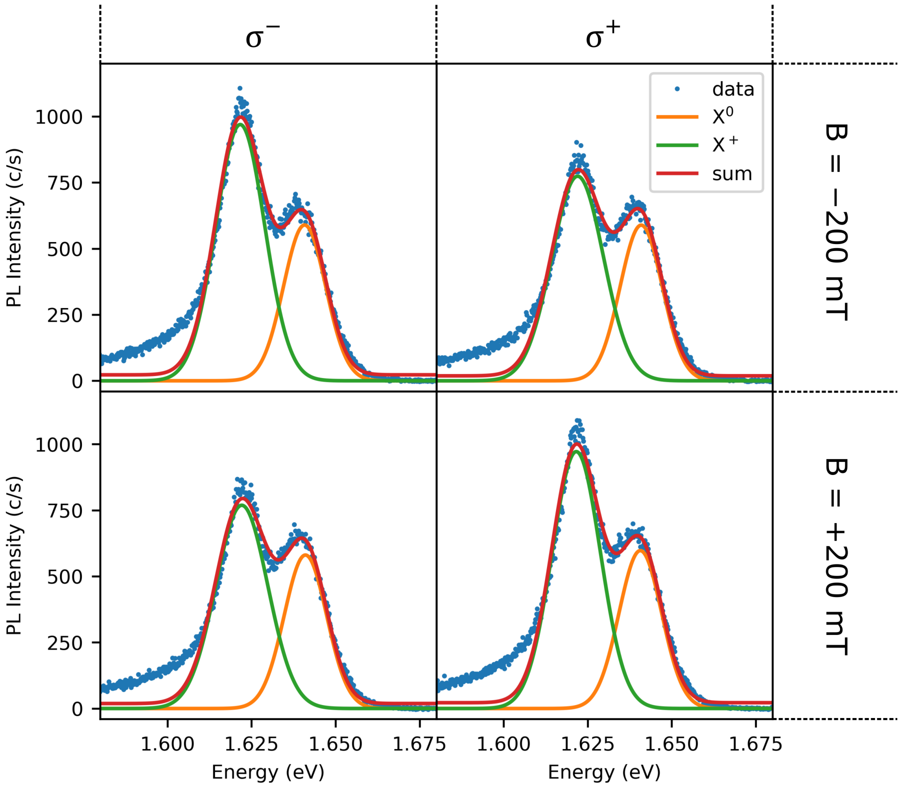

FIG. 8: Examples of photoluminescence spectra in both σ^+ and σ^- emission polarizations at external B-fields of ± 200 mT applied out of the sample plane. Each spectrum is fitted to two Gaussian peak functions.

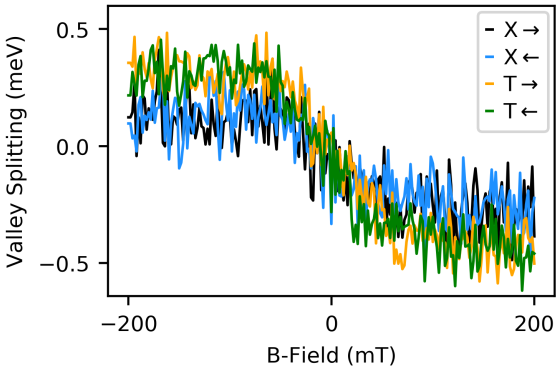

FIG. 9: Valley splitting of exciton (X) and trion (T) PL peaks extracted from the Gaussian peak fitting (both sweep directions).

We also fit the same spectra with 2 peaks: a higher energy symmetric Gaussian function for the neutral exciton, and a lower energy peak for the trion, which is the convolution between a Gaussian and a low energy exponential function, in order to attempt to account for the trion tail. Examples of the alternative fitting are shown below, along with the extracted standard deviation (a direct measure of FWHM in the exponentially modified Gaussian is not a well defined parameter, and so we use standard deviation instead to display how the peak width varies with B) and valley splitting vs B-field. As can be seen, the results are qualitatively in agreement with the double Gaussian peak fitting method shown above. The trion width switches between two values while the exciton remains almost constant (in agreement with Main Text Fig. 3c), while both peaks show a small valley splitting (in agreement with the valley splitting shown on the previous page).

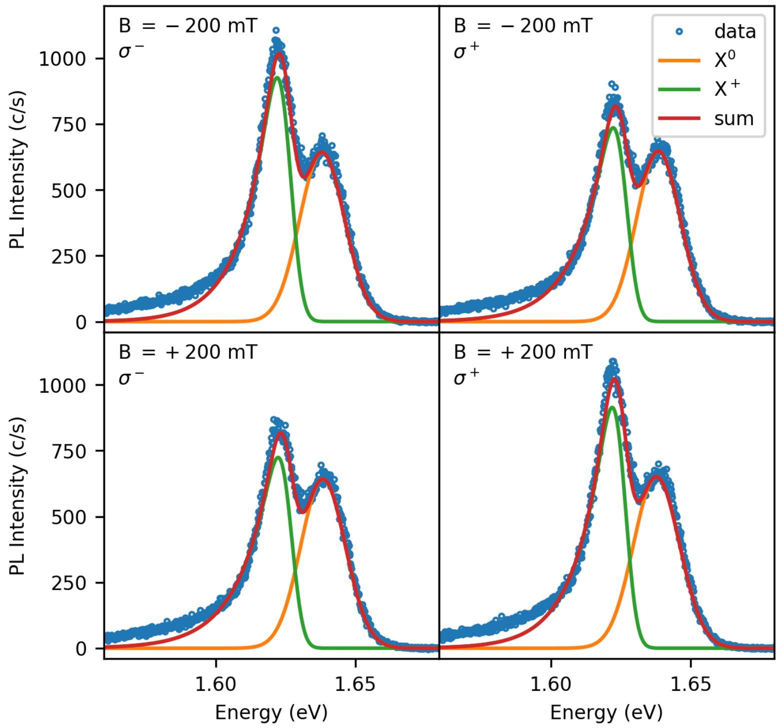

FIG. 10: Examples of peak fitting the spectra using a Gaussian for the exciton and a Gaussian convolved with an exponential function for the trion.

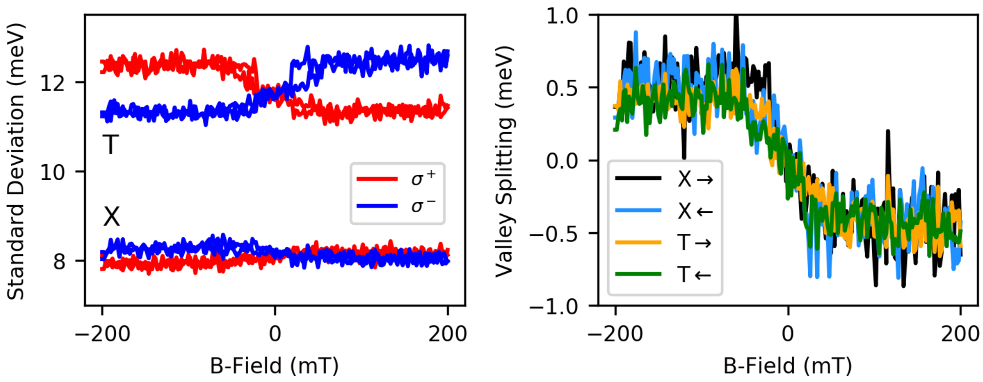

FIG. 11: Standard deviation (while not exactly equal to the FWHM, this gives a measure of peak width) and valley splitting of exciton (X) and trion (T) PL peaks obtained using the alternative peak fitting method.

Supplementary Note 7: The valley Zeeman effect

Throughout the Main Text we do not exceed 200 mT applied external B-field strength. This is to ensure that the valley Zeeman effect in $MoSe_2$ can be neglected, and so any observations must result purely from a magnetic proximity effect. The weak B-field serves only to control the domain dynamics in $CrBr_3$, and is too weak to have any appreciable effect directly on band energies in $MoSe_2$.

However, in order to investigate whether the $CrBr_3$ substrate modifies the valley Zeeman response of $MoSe_2$, we measure the valley splitting from the sample in applied fields up to $B = 8$ T. The result is shown below, where we additionally perform a linear fitting for both exciton and trion lineshifts to extract an average rate of shift. We find the exciton to shift at (-0.28 ± 0.01) meV / T while the trion shifts at (-0.11 ± 0.01) meV / T. Variation in rates of shift in $MoSe_2$ have been reported to be a consequence of doping level [12]. We also expect that $CrBr_3$, although saturated above $B = 200$ mT, may also modify the absolute rates of shift. In general, the exact nature of interplay between band shifts due to the valley Zeeman effect, and those due to interfacial exchange field, are not well understood.

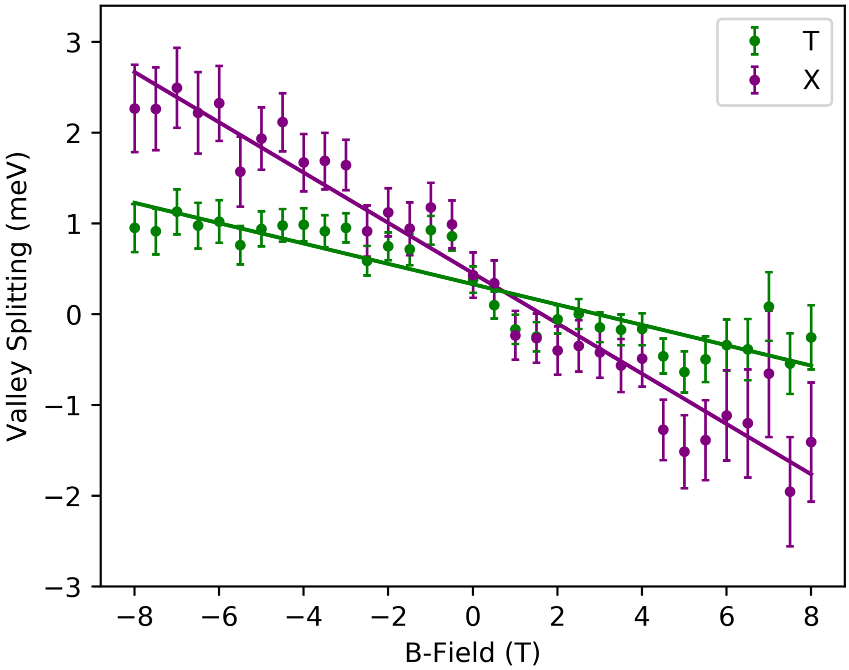

FIG. 12: Valley splitting of exciton (X) and trion (T) PL peaks in external magnetic fields up to $B = 8$ T. Solid lines are linear fits to the data.

Supplementary Note 8: Low temperature wide field Kerr microscopy

In addition to the magneto-photoluminescence measurements presented in the Main Text, the sample was studied using a low temperature wide-field Kerr microscope. Details of the wide field Kerr microscopy are given in the methods section. Despite storage under high vacuum and dark surroundings, significant degradation was observed to have occurred in the $CrBr_3$ flake in the period between completing the PL measurements and commencing the Kerr measurements. This most likely happened during sample transit in low vacuum, and during periods of sample mounting and unmounting from cryostats in air. Figure 13a shows the sample before the PL measurements, with the $CrBr_3$ flake intact. Figures 13b and c show the sample before Kerr measurements, after degradation.

Figures 13d, e, and f show images of the sample from the Kerr microscope at out of plane magnetic field strengths of $B = -80, 0, +80$ mT, and a sample temperature of 11 K. A background image, taken at zero applied field, is subtracted from the live CMOS feed, such that any magnetization manifests as brighter or darker regions of the image relative to the non-magnetic surroundings. This occurs as light reflected from magnetized material displays a small rotation of the linear polarization plane, and so is transmitted through the analyzer by a greater or lesser extent. In this way, the spatially resolved brightness of the image denotes the polar Kerr signal.

The brightness change of the remaining $CrBr_3$ is very clear, becoming darker than the surroundings at negative field and brighter at positive field. The change in brightness of the surrounding substrate is due to Faraday rotation in the microscope objective and cryostat window. Crucially, no domain structures could be resolved in the Kerr microscope at any field strength, rather, the entire flake appears to brighten and darken at the same rate. This indicates that the domains are smaller than the optical resolution of the microscope, ~ 300 nm. If they were larger, a mixed pattern of bright and dark regions would be visible in Figure 13e at zero field, but no such pattern is observed.

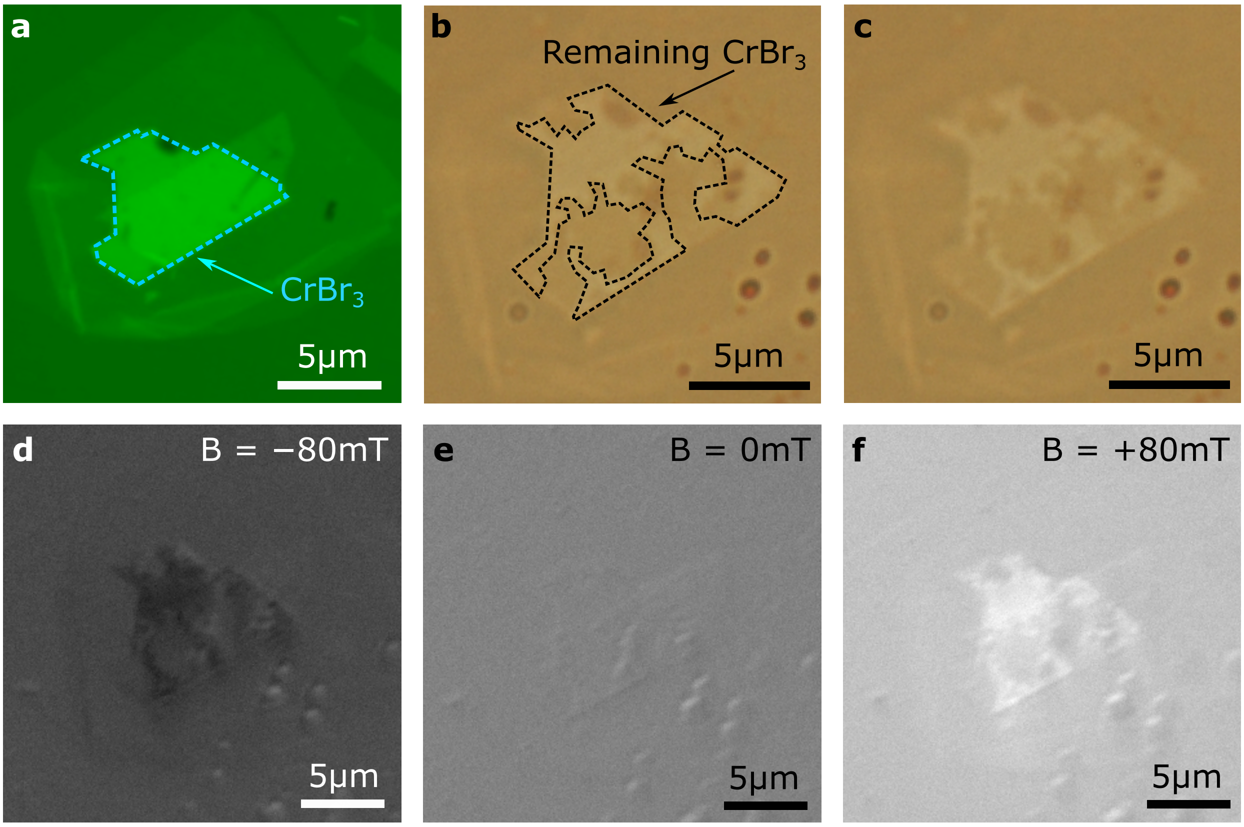

FIG. 13: Sample degradation and wide field Kerr microscopy. (a-c) Bright field microscope images of the sample (a) before the PL measurements presented in the Main Text, (b) before Kerr measurements, showing degradation of the flake, and (c) as (b) but without annotations for clarity. (d) Kerr microscope image of the sample under a magnetic field of $B = -80$ mT out of the plane. The dark $CrBr_3$ coresponds to a magnetization aligned to B. (e) Kerr microscope image at zero field. No domain structures can be resolved. (f) Kerr microscope image at $B = +80$ mT. The brightness of the flake indicates magnetization aligned to B. All Kerr microscopy is performed at a sample temperature of 11 K.

* Electronic address: t.lyons@sheffield.ac.uk
† Electronic address: a.tartakovskii@sheffield.ac.uk

[1] P. Giannozzi, S. Baroni, N. Bonini, M. Calandra, R. Car, C. Cavazzoni, D. Ceresoli, G. L. Chiarotti, M. Cococcioni, I. Dabo, *et al.*, *Quantum espresso: a modular and open-source software project for quantum simulations of materials,* Journal of Physics: Condensed Matter **21**, 395502 (2009).

[2] M. Wu, Z. Li, T. Cao, and S. G. Louie, *Physical origin of giant excitonic and magneto-optical responses in two-dimensional ferromagnetic insulators,* Nature Communications **10**, 2371 (2019).

[3] D. R. Hamann, *Optimized norm-conserving Vanderbilt pseudopotentials,* Physical Review B **88**, 085117 (2013).

[4] M. van Setten, M. Giantomassi, E. Bousquet, M. Verstraete, D. Hamann, X. Gonze, and G.-M. Rignanese, *The PseudoDojo: Training and grading a 85 element optimized norm-conserving pseudopotential table,* Computer Physics Communications **226**, 39 (2018).

[5] V. Popescu and A. Zunger, *Extracting E versus $\vec{k}$ effective band structure from supercell calculations on alloys and impurities,* Physical Review B **85**, 085201 (2012).

[6] A. Molina-Sánchez, K. Hummer, and L. Wirtz, *Vibrational and optical properties of MoS2: From monolayer to bulk,* Surface Science Reports **70**, 554 (2015).

[7] D. Ghazaryan, M. T. Greenaway, Z. Wang, V. H. Guarochico-Moreira, I. J. Vera-Marun, J. Yin, Y. Liao, S. V. Morozov, O. Kristanovski, A. I. Lichtenstein, *et al.*, *Magnon-assisted tunnelling in van der Waals heterostructures based on $CrBr_3$,* Nature Electronics **1**, 344 (2018).

[8] S. Dufferwiel, T. P. Lyons, D. D. Solnyshkov, A. A. P. Trichet, F. Withers, S. Schwarz, G. Malpuech, J. M. Smith, K. S. Novoselov, M. S. Skolnick, D. N. Krizhanovskii, and A. I. Tartakovskii, *Valley-addressable polaritons in atomically thin semiconductors,* Nature Photonics **11**, 497 (2017).

[9] G. Wang, E. Palleau, T. Amand, S. Tongay, X. Marie, and B. Urbaszek, *Polarization and time-resolved photoluminescence spectroscopy of excitons in $MoSe_2$ monolayers,* Applied Physics Letters **106**, 112101 (2015).

[10] M. Glazov, T. Amand, X. Marie, D. Lagarde, L. Bouet, and B. Urbaszek, *Exciton fine structure and spin decoherence in monolayers of transition metal dichalcogenides,* Physical Review B **89**, 201302 (2014).

[11] M. Z. Maialle, E. A. d. A. e Silva, and L. J. Sham, *Exciton spin dynamics in quantum wells,* Physical Review B **47**, 15776 (1993).

[12] Y. Li, J. Ludwig, T. Low, A. Chernikov, X. Cui, G. Arefe, Y. D. Kim, A. M. Van Der Zande, A. Rigosi, H. M. Hill, *et al.*, *Valley splitting and polarization by the Zeeman effect in monolayer $MoSe_2$,* Physical Review Letters **113**, 266804 (2014).
